# Open Reduction and Internal Fixation Versus Radial Head Arthroplasty for Mason III Radial Head Fractures: Appraising the Current Literature Evidence

**DOI:** 10.7759/cureus.7501

**Published:** 2020-04-01

**Authors:** Hannah Meacher, Shady Hermena, Sherif Isaac

**Affiliations:** 1 Trauma and Orthopedics, Royal Stoke Hospital, Newcastle Under Lyme, GBR; 2 Trauma and Orthopedics, Yeovil District Hospital, Yeovil, GBR; 3 Trauma and Orthopedics, Worcestershire Acute Hospitals National Health Service Trust, Worcester, GBR

**Keywords:** head radius fracture

## Abstract

Fractures of the radial head are common and account for one-third of elbow fractures. Management has evolved over the past few decades as have the techniques and implants used to treat them. However, no standardized treatment protocol exists because of the complexity with which these fractures may present. The complex, unstable, displaced, and multi-fragmentary fractures, also known as Mason type III fractures, remain one of the most challenging fractures to treat, especially if associated with other elbow injuries. There are various surgical treatment options available, including open reduction and internal fixation or radial head arthroplasty.

The purpose of this study was to systematically review the current literature that assessed open reduction and internal fixation compare to radial head replacement to identify the best surgical treatment protocol for the management of Mason type III radial head fracture.

All published clinical trials claiming to evaluate or cited elsewhere as being authoritative regarding the surgical treatment of radial head fractures were identified and evaluated. Studies in foreign languages (not in English) were excluded.

Based on two randomized controlled trials, this review showed some weak evidence that arthroplasty results in better functional elbow outcomes and lower complication rates as compared to open reduction and internal fixation. There is a scarcity of good quality comparative studies and multicenter randomized controlled trials should be considered.

## Introduction and background

The radial head is an important structure and is crucial to elbow and forearm stability. It also contributes significantly to forearm rotation. Fracture of the radial head typically occurs after a fall onto an outstretched hand. It accounts for 4% of all fractures and nearly one-third of elbow fractures [1-2]

In 1954, Mason classified radial head fractures into three simple types: type 1 undisplaced fractures, type 2 displaced partial head fractures, and type 3 displaced fractures that involve the entire radial head [2]. This classification has been modified several times because of its limitations in addressing stability and associated elbow injuries, such as radial neck fractures and ligamentous injuries, and their implications on treatment and functional outcome. However, due to the ease of use in daily practice, it remains widely used [3-8].

The management of Mason type III fractures remains controversial and continues to challenge the treating surgeon for several reasons. These fractures are displaced, multi-fragmentary and intra-articular and, therefore, anatomical reconstruction is mandatory for good functional outcomes. They are also associated with other elbow injuries that need to be addressed at the time of treatment [8-15]. In addition, the dynamics of the native radial head contribute to elbow stability and forearm rotation [16].

Historically, the treatment of displaced comminuted radial head fractures primarily involved excision of the radial head, which leads to instability, restriction of motion, and distal radio-ulnar joint dysfunction [16-18]. Therefore, with the development of advanced internal fixation and arthroplasty techniques and the increased appreciation of the important contribution of the radial head to forearm and elbow stability, radial head excision is rarely used nowadays [19-23]. An exception to this would be in the context of a terrible triad injury where data suggest there is little difference in outcome for patients undergoing excision or arthroplasty [24]. Currently, the two main surgical treatment options for Mason type III fractures are open reduction and internal fixation or radial head replacement with a metal prosthesis, each with its successful cases and flaws.

The aim of this study was to review the literature and identify which one of these two surgical treatment options has a better outcome.

## Review

Methods and materials

We followed the guidelines for Preferred Reporting Items for Systematic Reviews and Meta-Analyses (PRISMA) [25]. 

Eligibility

Studies that met the following criteria were identified:

i) Target population: Adult patients with Mason III radial head fracture regardless of the mechanism of injury and the presence of associated elbow injuries.

ii) Intervention: Surgical treatment with open reduction and internal fixation or radial head arthroplasty using a metal prosthesis.

iii) Comparison: Clinical trials that compared open reduction and internal fixation to radial head arthroplasty.

iv) Methodology: Published clinical comparative trials and randomized or quasi-randomized studies were included in the review.

Exclusion Criteria

Studies that used a Silastic head prosthesis were excluded. Studies that are in a foreign language (not in English) were also excluded.

Study Identification

Relevant studies published between January 1946 and January 2019 were identified using MEDLINE, Embase, Allied and Complementary Medicine Database (AMED), Cumulative Index to Nursing and Allied Health Literature (CINAHL), PubMed, International Political Science Abstract, and the Cochrane database. The MEDLINE and Embase search was done on OvidSP (available at http://ovidsp.tx.ovid.com/sp-3.5.1a/ovidweb.cgi). A search set was created using the terms: “radial head.” A search strategy was then built by adding the terms fracture and internal fixation in isolation and combined. Another search strategy was created by adding the terms arthroplasty and replacement in isolation and/or combined with radial head and fractures.

Data Extraction

The authors extracted all the relevant information regarding the population, intervention, study methodology, and functional outcome, including complications.

Outcome Measures

The primary outcome measure was the functional outcome, which included the range of motion, stability, and pain. The secondary outcome measure was the development of complications.

Results

**Figure 1:**
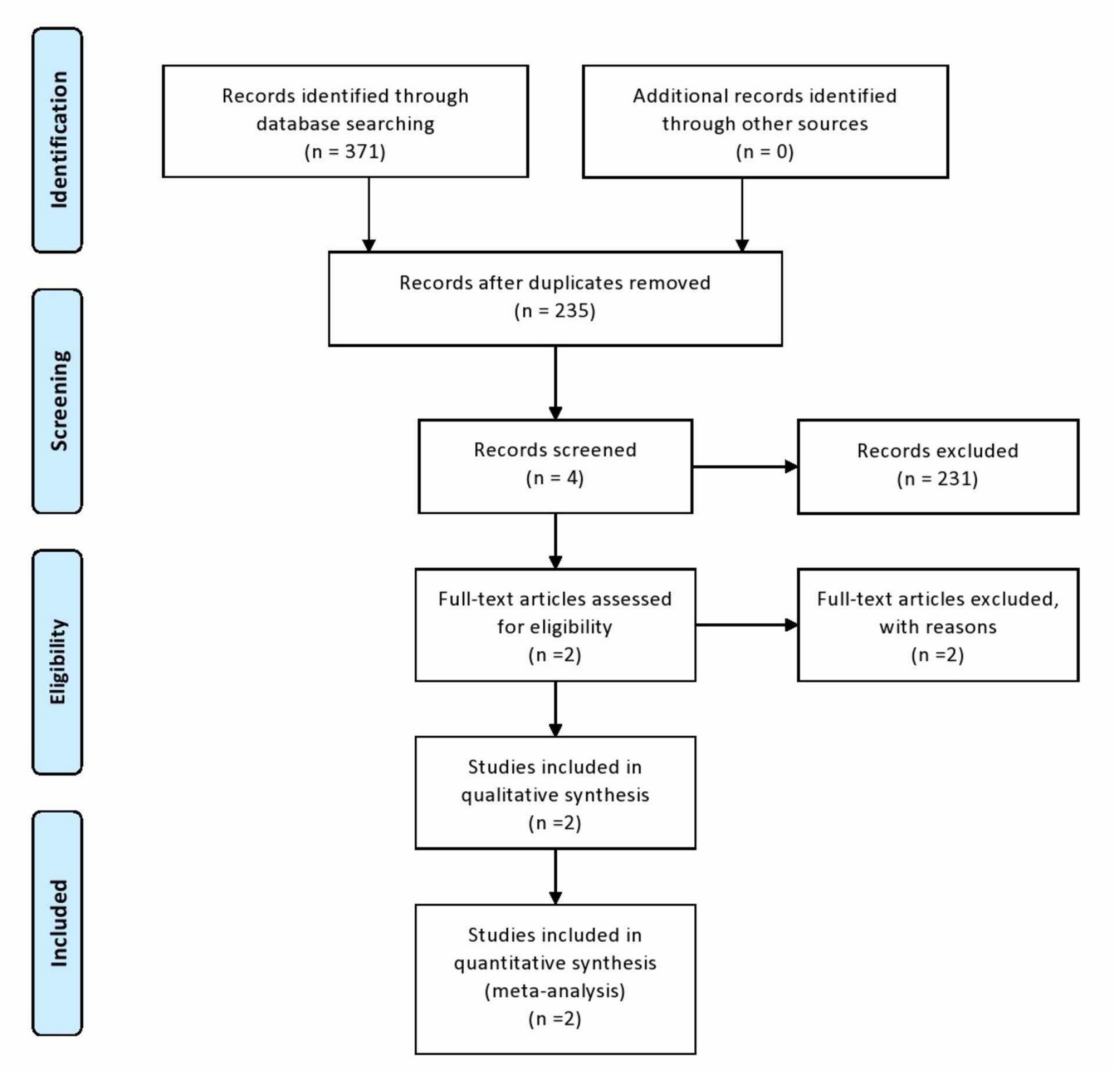
PRISMA flow of the search results and selection procedure PRISMA: Preferred Reporting Items for Systematic Reviews and Meta-Analyses

The computerized database search is shown in the appendix. A total of 371 articles that dealt with radial head fractures were identified at the conclusion of the search strategy. These articles were reviewed by the authors and examined against the inclusion criteria. There were two articles that were randomized controlled trials and met the inclusion criteria and, therefore, were included in this review [26-27]. A further article discussed open reduction and internal fixation versus arthroplasty in radial head fractures but was excluded as the article is in the Chinese language and hence unable to be appraised [28]. Another article that dealt with the biomechanical evaluation of the elbow joint following open reduction and internal fixation or Silastic radial head replacement was also excluded as the prosthesis used was not metal and, therefore, did not meet the inclusion criteria [29]. No further articles were identified by manual search through the references of the recovered articles. Figure 1 shows the Prisma flow chart for the research process.

In 2009, Ruan et al. published a comparative study of internal fixation and prosthesis replacement for Mason type III radial head fractures [26]. Despite the fact that this study was a prospective randomized trial, the authors failed to demonstrate how the randomization was conducted and, consequently, this might question their conclusion. There were 22 patients included in the study. Fourteen patients underwent radial head replacement and eight patients had an internal fixation. This is a small sample size, especially in the fixation group, and, therefore, the author’s conclusion might not be accurate due to a type II error. There was no statistical difference between the two groups regarding age and time to surgery, despite there being two patients in the replacement group who were more than 12 months post-injury before they were operated on. All patients consented to the study but there was no evidence that ethical approval for the study has been sought. Postoperatively, the replacement group patients started passive rehabilitation after 48 hours while the internal fixation group patients remained in plaster for four weeks before physiotherapy started. This could be an advantage of arthroplasty over internal fixation that would contribute to a better range of elbow motion. Follow-up was under 16 months for the replacement group (range 10 - 27 months) as compared to 14 months (range 10 - 21 months) in the fixation group. This is a short follow-up period considering the small number of patients in each group to conclude. However, it might be difficult to follow up patients in the fixation group any longer because most of these patients had a failure of fixation and might have undergone further treatment despite the fact that this was not clearly demonstrated in the study. The authors used the elbow functional evaluation criteria by Broberg and Morrey [30]. This is an established functional evaluation score for elbow function following trauma. This score was first developed and used by Broberg and Morrey in 1986 to assess elbow function following delayed excision of the radial head following a fracture [31]. Reviewing the literature, this elbow score has not been validated even though it is widely used to assess elbow function in a variety of conditions, including trauma and arthritis [18,30,32-33]. The score ranges from 0 to 100 based on four categories, which are the range of motion, grip strength, functional stability, and pain. This then determines whether the score is Excellent, Good, Fair, or Poor. The outcome results were statistically significant in favor of radial head replacement.

The complication rate was also higher in patients in the internal fixation group. The authors did not demonstrate any associated elbow injuries, which are common with type III fractures and their incidence as well as distribution among the two groups [9-10]. This could have an impact on the outcome. The functional outcome after treating an isolated radial head fracture would be different when there are associated injuries such as the terrible triad [8-15]. Based on their results, the authors claimed that radial head arthroplasty is a better option for Mason type III fractures as compared to open reduction and internal fixation.

In 2011, Chen et al. conducted a prospective randomized controlled trial comparing radial head replacement and open reduction and internal fixation for Mason type III fractures [26]. The patients were randomized to one of the two treatment options but, yet again, the authors did not explain the method of randomization. There were 45 patients included with 22 in the arthroplasty group and 23 in the fixation group. This is a reasonable number of patients that might result in a valuable conclusion. Despite randomization, the authors did not demonstrate if there was equal distribution regarding age and sex between the two groups, as this might have an effect on the interpretation of the study result. Furthermore, the authors included all Mason type III fractures even if associated with other elbow injuries but failed again to declare how many patients with associated elbow injuries were in each group. It is known that the more the severity of the injury, indicated by other associated bony or soft tissue elbow injuries, the less favorable outcome is achieved. All patients consented to the study and ethical approval was obtained. There were clear inclusion and exclusion criteria. However, the authors excluded patients with severely comminuted radial head fractures and, by definition, according to Mason classification, type III is where the radial head is comminuted [2]. Nevertheless, the authors did not give a definition, in their opinion, to a severely comminuted radial head fracture. This would directly lead to bias as the authors might have excluded the patients they knew that they would perform poorly with any of the treatment options, type I error. A radial head replacement was performed by one surgeon but it was not clear if this was the case in the internal fixation group. Postoperatively, the radial head replacement group started active exercises within a week unless there were associated injuries when the patients were immobilized for three weeks. In the internal fixation group, patients started exercises after a period of immobilization for four weeks. This postop regime favors the arthroplasty surgical option from the range of motion point of view. Mean follow-up was just under three years (range 1 - 5 years) with no loss of follow-up. At follow-up, all the reviewers were blinded to the method of treatment. This is essential to minimize bias toward a specific type of treatment. Patients were assessed according to the Broberg and Morrey functional elbow score [31]. The score in the arthroplasty group was better than the internal fixation group and this was statistically significant. The complication rate was also recorded in both groups and this was significantly better in the arthroplasty group, with only three patients developing a complication as compared to 11 patients in the internal fixation group. The authors concluded, based on their results, that radial head arthroplasty with a metal prosthesis resulted in a favorable elbow function and a less complication rate as compared to open reduction and internal fixation in the treatment of unstable multi-fragmented fractures of the radial head.

Discussion

Radial head fractures occur in 17% - 19 % of cases of elbow trauma and account for 33% of elbow fractures [34]. The treatment of Mason type III fractures is controversial. Early treatment included radial head excision but due to a high complication rate, this option is rarely indicated [20-23]. Radial head excision can lead to distal radioulnar joint arthritis, elbow instability, increased elbow valgus, which might lead to ulnar nerve neuropathy symptoms and reduction in elbow extension [17-18,20,26,34-35]. The other surgical treatment options are open reduction and internal fixation or radial head arthroplasty.

The radial head is essential for elbow biomechanics [17,20-23]. It is the primary restraint to the proximal migration of the radius and, therefore, contributes to elbow and forearm stability [36]. It is also important for forearm rotation and elbow flexion and extension. Therefore, attempts to maintain the radial head by open reduction and internal fixation was a popular treatment option with satisfactory results [30,32-33,37-39]. The satisfactory early reports of internal fixation were due to focusing primarily on isolated fractures that involved only part of the radial head [40]. Subsequent reports showed that this option is prone to failure due to non-union, loosening of the fixation device, restricted forearm rotation, and elbow stiffness, especially if the radial head is fractured in three or more fragments [41-42]. The associated injury to the lateral ulnar collateral ligament and ulnar collateral ligament complex will result in instability if the radial head is excised and the ligaments are not repaired [36]. Similarly, injury to the interosseous membrane, as in Essex- Lopresti injuries, will lead to longitudinal forearm instability if the radial head is not replaced [11]. Consequently, researches tried to find an alternative option and, therefore, radial head arthroplasty has become a popular option in recent years.

The disadvantage of a radial head arthroplasty is inserting the prosthesis that is too long, which can lead to subluxation of the elbow and capitellar wear [43-44]. In addition, the anatomy of the native radial head is complex and difficult to replicate. It is elliptical in shape and angled at 15 degrees to the radial shaft, and yet there is no available radial head prosthetic implant that matches the exact shape and reproduces its dynamics [16,45-46]. In 2004, Beingessner et al. studied the biomechanical effects of radial head excision and prosthetic arthroplasty on elbow kinematics in a cadaver model [47]. It has become clear that radial head replacement improves stability but still does not return the elbow to its normal function.

Mazhar et al. performed a retrospective comparative cohort study comparing resection versus arthroplasty in the context of a terrible triad injury, finding a little difference in patients receiving either treatment [24].

## Conclusions

The aim of this review was to assess the current literature evidence that compared these two surgical treatment options for Mason type III radial head fractures. There were only two randomized trials that were published recently in the same journal that compared internal fixation versus arthroplasty with the same conclusion. The first study by Ruan et al. is not a well-designed randomized trial and its interpretation should be considered with caution. The second study was a better-randomized trial with some flaws but yet gave some evidence that arthroplasty of the radial head is superior to open reduction and internal fixation. Meta-analysis was not feasible to perform not only because there are only two randomized controlled trials but also because the qualitative representation of the Broberg and Morrey score made it impossible to combine overall figures from the two trials. Satisfactory treatment of Mason type III fractures could be achieved with radial head arthroplasty and may be a better option to choose when treating these complex fractures but the current evidence is weak based on this review. Therefore, this review should stimulate researchers to set up a multicentre prospective randomized trial with an appropriate sample size to compare treatment options in the management of these common complex fractures.

## Appendices

**Table 1:** Computerized database search

	Searches	Results
1	radial head.mp. [mp=ab, hw, ti, ot, nm, ps, rs, ui, tx, bt, ct, sh, tn, dm, mf, dv, kw, an, au, jn, tt]	6678
2	fracture.mp. [mp=ab, hw, ti, ot, nm, ps, rs, ui, tx, bt, ct, sh, tn, dm, mf, dv, kw, an, au, jn, tt]	540148
3	internal fixation.mp. [mp=ab, hw, ti, ot, nm, ps, rs, ui, tx, bt, ct, sh, tn, dm, mf, dv, kw, an, au, jn, tt]	50788
4	1 and 2 and 3	1563
5	arthroplasty.mp. [mp=ab, hw, ti, ot, nm, ps, rs, ui, tx, bt, ct, sh, tn, dm, mf, dv, kw, an, au, jn, tt]	156473
6	replacement.mp. [mp=ab, hw, ti, ot, nm, ps, rs, ui, tx, bt, ct, sh, tn, dm, mf, dv, kw, an, au, jn, tt]	813356
7	1 and 2 and 5	1051
8	1 and 2 and 6	1132
9	7 or 8	1568
10	7 and 8	615
11	4 and 7 and 8	371
12	4 and 9	818
